# Congenital adrenal hyperplasia patient perception of ‘disorders of sex development’ nomenclature

**DOI:** 10.1186/s13633-015-0004-4

**Published:** 2015-03-16

**Authors:** Karen Lin-Su, Oksana Lekarev, Dix P Poppas, Maria G Vogiatzi

**Affiliations:** Department of Pediatric Endocrinology, Weill Cornell Medical Center, 505 East 70th Street, 3rd floor, New York, NY 10021 USA; Department of Pediatric Urology, Weill Cornell Medical Center, 525 East 68th Street, Suite F-943, New York, NY 10065 USA

**Keywords:** Congenital adrenal hyperplasia, Disorders of sex development, Nomenclature

## Abstract

**Background:**

As the benefits of patient-centered care have become more widely recognized, it is important to understand patients’ sentiments regarding aspects affecting their care. In an effort to display more sensitivity to patient concerns, the term “disorders of sex development” (DSD) was proposed in 2006 as new nomenclature to replace older terms that were considered to have negative connotations.

**Methods:**

The objective of the study was to examine the views of congenital adrenal hyperplasia (CAH) patients and their caregivers regarding the new nomenclature. The study was observational to evaluate the views of the CAH community, and the primary endpoint was perception of the term DSD. The study was conducted as a survey about views regarding DSD nomenclature. The survey was sent via email to eligible subjects. Along with a short introduction explaining the term DSD, the survey was sent to eligible CAH patients and their caregivers. 589 CAH patients or family members participated in the survey.

**Results:**

A total of 589 responses were received (255 classical females, 104 non-classical females, 174 males, 56 not specified) (547 U.S., 42 international) (128 CAH patients, 408 parents or other family members). 70.6% had never heard the term DSD. 71.0% disliked or strongly disliked the term DSD. 83.6% stated they did not identify with the term DSD. 76.0% felt that the term DSD has a negative effect on the CAH community. There was no significant difference in opinion of DSD between classical females and other CAH patients, between US and international, between surgical and non-surgical patients, or between patients and parents. There was no correlation with patient age.

**Conclusions:**

Our results indicate that the majority of parents and patients with CAH are dissatisfied with the term DSD. Our results highlight the challenges within the field of DSD to reach a consensus regarding a sensitive topic and to bridge the gap between current medical practice and patient satisfaction. It is the authors’ belief that reconsideration of the current nomenclature and ongoing dialogue between the medical community and patients will eventually lead to removal of stigmatization, better management protocols, and improved outcomes.

## Background

Disorders of sex development (DSD) is an umbrella term that was proposed by the International Intersex Consensus Conference in 2006 to describe conditions in which fetal development of chromosomal, gonadal, or anatomic sex is atypical [1]. Due to concerns raised by patient advocacy groups, the term DSD was developed to replace previously employed terms, such as “intersex”, “pseudohermaphroditism”, “hermaphroditism”, “sex reversal”, and other labels that were perceived by patients and parents to have negative connotations [2-4]. A study by Pasterski et al. (2010) found that 100% of the pediatric endocrinologists surveyed in Europe reported using the new terminology [5], indicating that the medical community is responsive to patient views and receptive to evolving terminology. However, data regarding patient perception of the new nomenclature are limited.

Examples of medical conditions included under the term DSD are: 46,XX classical 21-hydroxylase deficiency (21OHD) congenital adrenal hyperplasia (CAH), androgen insensitivity syndrome (partial or complete), gonadal dysgenesis, ovotesticular DSD, and 5-alpha-reductase deficiency. Due to the extreme rarity of many of these conditions, 46,XX classical 21OHD CAH accounts for a substantial portion of cases of those grouped under the term DSD. CAH is a family of autosomal recessive enzyme deficiencies that cause insufficient cortisol production by the adrenal cortex; 21OHD accounts for >90% of CAH cases. In 21OHD CAH, cortisol deficiency leads to increased ACTH production and subsequent excess androgen production by the adrenal cortex [6]. Females with the classical (severe) form of CAH are born with varying degrees of androgenized genitalia and are included under the term DSD. Males with 21OHD CAH and females with the non-classical (mild) form are born without any effects on their genitalia, thus are not included under DSD.

The new term DSD was introduced with the hope of developing terminology that would be more sensitive to patient views while at the same time maintaining scientific and medical integrity. A small study in 2010 evaluated acceptability of the new terminology among parents and health professionals; however, the sample included only 19 parents of children with a DSD [7]. As the term DSD has become more widely utilized in the literature, internet, research proposals, and patient care clinics, parents and patients with CAH have expressed their concerns to the authors about the potential misconceptions that could arise from having CAH linked to the words “sex” and “disorder”. It is important, therefore, to understand the new nomenclature’s perceived connotation and its impact on patients and families.

The goal of the study was to determine perception of the CAH community towards the new term DSD. To achieve this objective, the authors surveyed CAH patients and their caregivers. Subjects were recruited through CARES (**C**ongenital **A**drenal hyperplasia **R**esearch **E**ducation and **S**upport) Foundation, a U.S.-based (with international representation) patient advocacy group for CAH patients and their families.

## Methods

### Subjects

Potential subjects were identified through CARES Foundation’s online database, which includes email addresses for all registered patients and families. Inclusion criteria were: 1) CAH patient or family member of a patient with CAH, 2) at least 18 years of age, and 3) able to read and understand English. Email contacts were excluded if they were incomplete, duplicated, or from the same household.

### Survey

A short introduction explaining the term DSD along with a questionnaire regarding nomenclature were sent via email using Survey Monkey; only one survey was sent per household. Survey responses were anonymous. Approval was obtained from the Institutional Review Board at Weill Cornell Medical Center.

Questions from the survey included:

1. *Have you ever heard the term “disorder of sex development” or DSD?*
2. *When is the first time you heard the term DSD used to describe CAH?*
3. *What do you think of the term DSD?*
4. *Do you identify with the use of the term DSD as a CAH parent or patient?*
5. *How do you feel about clinics that monitor patients with CAH and other conditions being called DSD clinics?*
6. *Would you seek care for yourself or your child at a clinic or center that used the term DSD to encompass CAH?*
7. *Would you participate in research studies using the term DSD instead of CAH?*
8. *In your opinion, what effect does using the term DSD have on the CAH community?*
9. *How do you feel about the term “ambiguous genitalia” to describe genitalia that are not definitively male or female?*
10. *If you would rather have an alternate way of describing genitalia at birth that are not definitively male or female, what term(s) do you prefer?*

### Statistical analysis

The primary endpoint variable was opinion regarding the term DSD (5 point scale: 1 = strongly dislike; 2 = dislike; 3 = neutral; 4 = don’t mind; 5 = like). A secondary endpoint variable was opinion regarding the term “ambiguous genitalia” (5 point scale as above). A student’s t-test was used for comparisons of mean opinion scores between two groups (classical females vs. other CAH patients; parents vs. patients). A Welch’s t-test was used for comparisons between U.S. vs non-U.S and between surgical vs non-surgical classical females. Analysis using Pearson’s correlation coefficient was performed to assess the relationship between patient age and opinion. A chi square test of independence was performed to examine differences between groups with categorical variables (yes/no). A result was considered to be statistically significant if *p* <0.05.

## Results

### Participants

The survey was sent via email to 2032 eligible U.S. CAH families and 254 eligible international CAH families. A total of 547 U.S. responses and 42 non-U.S. responses were received. When questions were left unanswered, these numbers were excluded from statistical analysis.

Most of the survey takers were parents of CAH patients. Of 534 people who described their relationship to the patient, 128 (24.0%) were CAH patients, 374 (70.0%) were parents, and 32 (6.0%) were other relatives of patients, e.g. grandparent, aunt/uncle, or sibling. Sixty-six (12.4%) were parents of more than one affected child, 10 (1.9%) were parents of affected children and patients themselves, and 4 (0.7%) were patients who had affected siblings.

The majority of patients had classical CAH: 406 (76.5%) patients had classical CAH, and 125 (23.5%) had non-classical CAH. The mean patient age at the time of the survey was 17.6 ± 15.5 years (range 2 months to 70 years). Median patient age was 12 years (interquartile range = 6 to 26 years).

Out of 537 responses, 359 (66.9%) patients were female (255 classical; 104 non-classical), 174 (32.4%) were male, and 4 (0.7%) answered “other”, e.g. more than one affected or “not relevant.”

Of the 255 classical female patients, 30 neither had nor planned to have genital surgery (non-surgical); 3 had received prenatal treatment. 223 classical females either had genital surgery or planned to have surgery (surgical).

### Awareness of the term DSD

**Figure 1:**
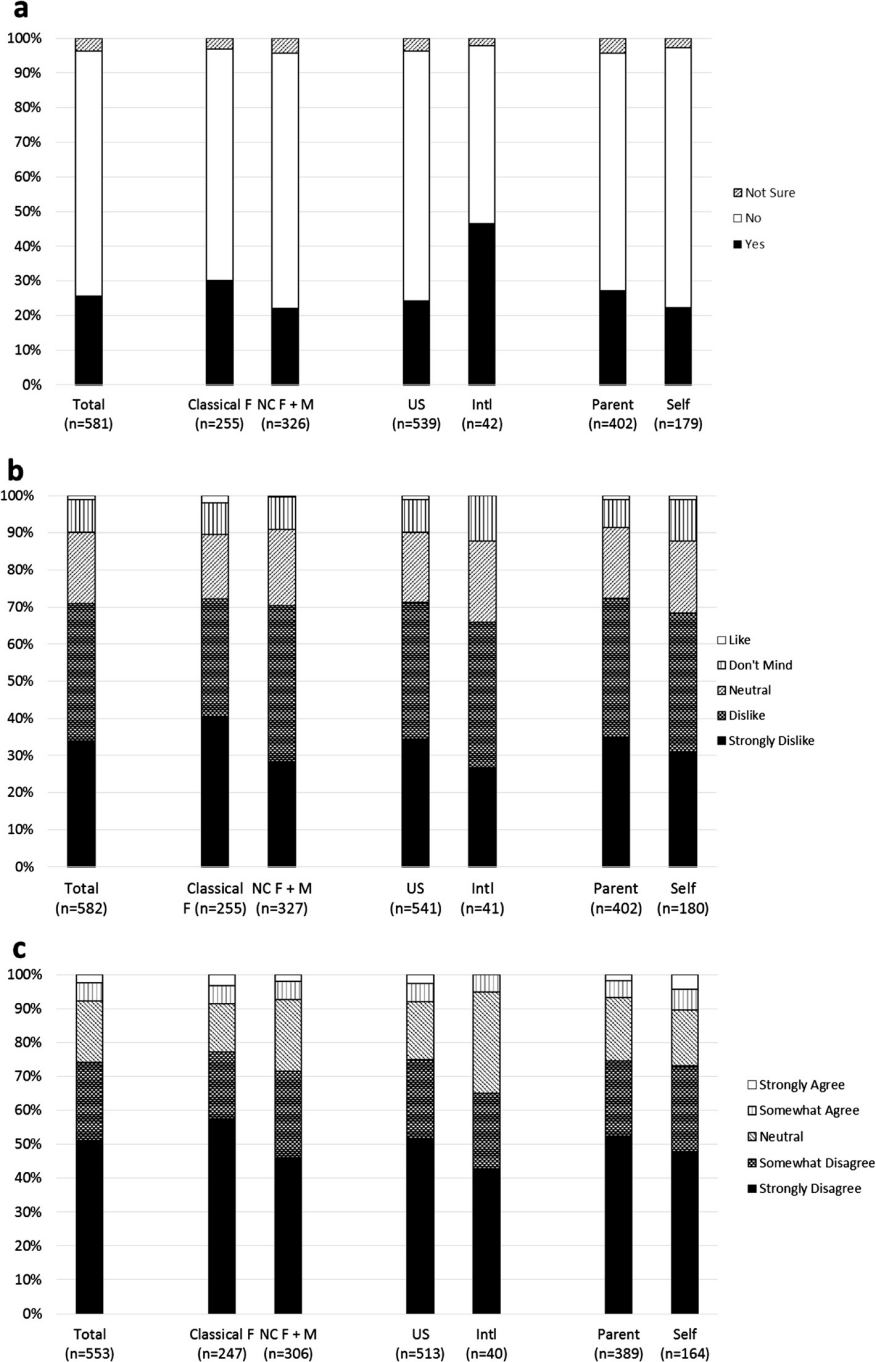
**Response percentages regarding DSD. a**: Have you ever heard the term “disorder of sexual development” or DSD? **b**: What do you think of the term DSD? **c**: How do you feel about clinics that monitor CAH patients being called DSD clinics? Classical F = female patients with classical CAH; NC F + M = female patients with non-classical CAH and male patients with any form of CAH. US = United States; Intl = International. Parent = survey completed by the parent or family member of CAH patient; Self = survey completed by the CAH patient

Of 581 survey takers, 149 (25.6%) had previously heard the term DSD, 410 (70.6%) had never heard the term before, and 22 (3.8%) were not sure. Compared to U.S. survey takers, non-U.S. survey takers were more likely to have heard the term DSD, (24.1% vs. 45.2% respectively, *Х*^2^ = 9.57, *p* <0.01) (see Figure 1a). Of those who were previously aware of the term, 80 (53.7%) heard it when researching the internet, 29 (19.4%) at a conference, 27 (18.1%) when their child was born, and 6 (4.0%) at the first post-diagnosis appointment.

### Opinion regarding DSD nomenclature

When asked what they think of the term DSD, 196 (33.7%) strongly dislike it, 217 (37.3%) dislike it, 111 (19.1%) are neutral, 52 (8.9%) don’t mind it, and 6 (1.0%) like it (see Figure 1b). There was no correlation between patient age and opinion regarding the term DSD: *r* = 0.02, *p* = 0.64.

There was no statistical difference in mean opinion scores between classical females and other CAH patients, between parents/family members and patients, between U.S. and non-U.S. participants, or between surgical and non-surgical classical females (see Table 1).

**Table 1:** **Opinion scores regarding the terms “DSD” and “ambiguous genitalia”**

*What do you think of the term DSD?*	Classical females vs Other CAH	2.0 ± 1.0 vs 2.1 ± 0.9
	Parents vs Patients	2.0 ± 1.0 vs 2.2 ± 1.0
	US vs non-US patients	2.0 ± 1.0 vs 2.2 ± 1.0
	Surgical vs non-surgical	2.0 ± 1.0 vs 2.1 ± 1.2
*What do you think of the term ambiguous genitalia?*	Classical females vs Other CAH	3.0 ± 1.1 vs 3.4 + 0.9*
	Parents vs Patients	3.2 ± 1.1 vs 3.1 ± 1.0
	US vs non-US patients	3.2 ± 1.1 vs 3.0 ± 1.2
	Surgical vs non-surgical	3.0 + 1.1 vs 3.0 + 1.2

1 = Strong dislike; 2 = Dislike; 3 = Neutral; 4 = Don’t mind; 5 = Like.

Results are expressed as mean ± SD.

*p <0.01.

Of 574 responses, 480 (83.6%) said they do not identify with the term DSD as either a CAH parent or patient; 94 (16.4%) said they identify with the term DSD.

### Views on clinics/centers and research using the term DSD

When asked how they feel about clinics that monitor CAH patients being called DSD clinics, 282 (51.0%) strongly disagree, 128 (23.1%) somewhat disagree, 100 (18.1%) are neutral, 29 (5.2%) somewhat agree, and 14 (2.5%) strongly agree (see Figure 1c).

When asked if they would seek care at a clinic or center that used the term DSD to encompass CAH, 286 (49.5%) said *No*, 198 (34.2%) said *Maybe*, and 94 (16.3%) said *Yes*.

Regarding participation in research studies that use the term DSD instead of CAH, 310 (53.2%) said *No*, 200 (34.3%) said *Maybe*, and 73 (12.5%) said *Yes*.

### Impact of DSD on the CAH community

**Figure 2:**
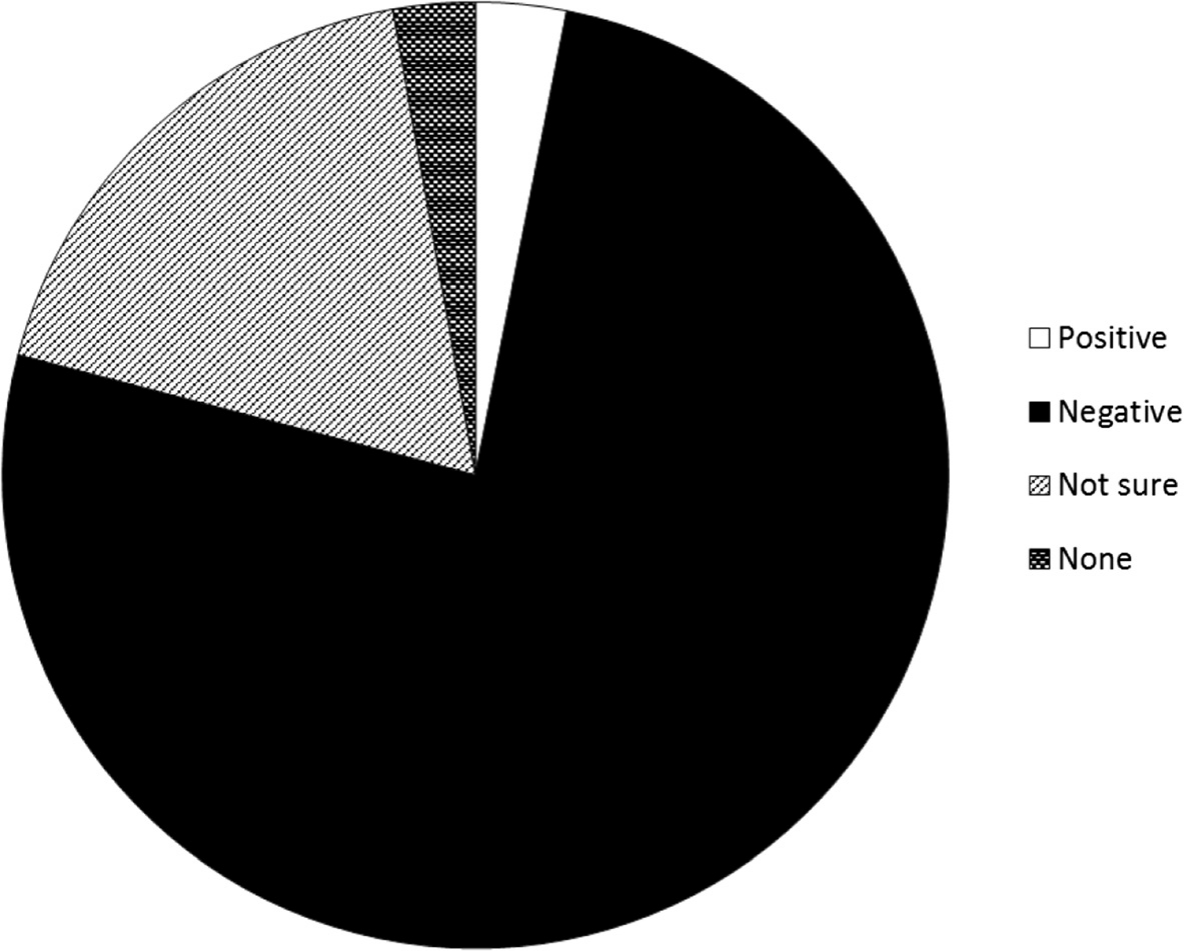
**In your opinion, what effect does using the term DSD have on the CAH community?** (n = 580).

Out of 580 responses, 441 (76.0%) believe that using the term DSD has a negative effect on the CAH community; 105 (18.1%) are not sure; 18 (3.1%) feel it has a positive effect; 16 (2.8%) feel that it has no effect (see Figure 2).

### Opinion regarding the term ambiguous genitalia

**Figure 3:**
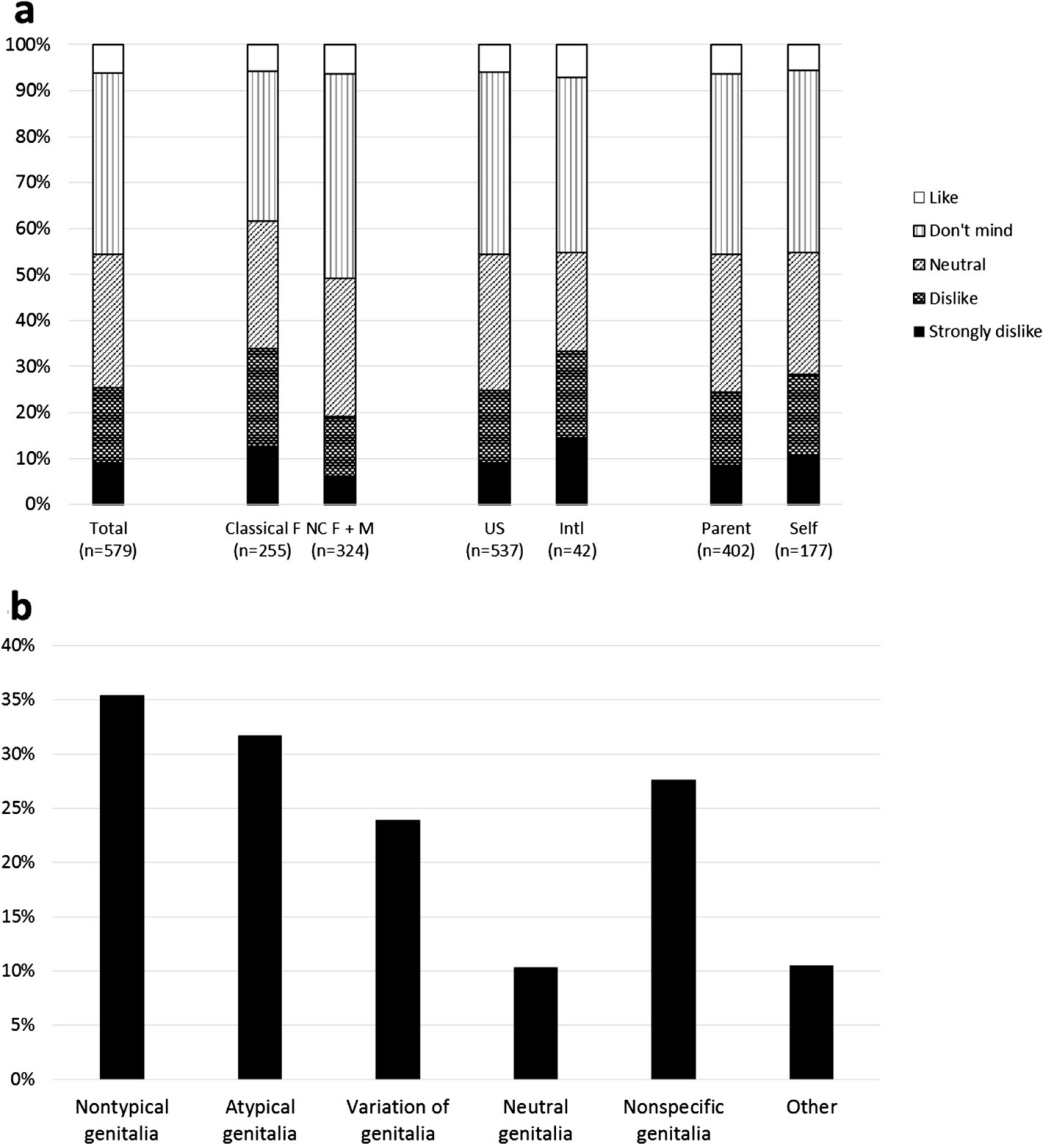
**Response percentages regarding ambiguous genitalia. a**: How do you feel about the term ‘ambiguous’ to describe genitalia at birth that are not definitively male or female? **b**: If you would rather have an alternate way of describing genitalia at birth that are not definitively male or female, what term(s) do you prefer? Please check all that apply.

When asked what they think of the term ambiguous genitalia, 52 (9.0%) strongly dislike it, 95 (16.4%) dislike it, 168 (29.0%) are neutral, 228 (39.4%) don’t mind it, and 36 (6.2%) like it (see Figure 3a). The mean opinion score of classical females (3.0 ± 1.1) was significantly lower than the mean opinion score of other CAH patients (3.4 ± 0.9), *p* <0.01. There was no significant difference between parents and patients, between U.S. and non-U.S., or between surgical and non-surgical classical females (see Table 1).

There was no correlation between patient age and opinion regarding the term ambiguous genitalia: *r* = 0.01; *p* = 0.82.

There were 486 participants who selected alternate terms to describe genitalia at birth that are not definitively male or female (see Figure 3b), with the highest percentage (35.4%) preferring *nontypical* genitalia.

## Discussion

This report describes the views of CAH patients and their caregivers regarding DSD nomenclature. Although the term was developed with the goal of being more sensitive to affected individuals, those represented in this survey feel that the term has a negative impact on the CAH community. As the benefits of patient-centered care are becoming more widely recognized [8-10], it is important to understand patients’ thoughts and sentiments regarding aspects affecting their care. This study is of great significance because it provides the first comprehensive survey revealing the perception of CAH patients towards an extremely sensitive topic.

In the earlier study by Davies et al. (2010), nearly 95% of parents preferred the new term over “intersex”, but only 37% considered DSD to be an acceptable replacement term [7]. Similarly, our results indicate that only 10% of participants had a favorable opinion of the term DSD, whereas over 70% disliked or strongly disliked it. The finding that only 26% had heard the term DSD prior to the survey (and only a small minority first heard it from a medical professional) may suggest that health care providers are not routinely using the term when communicating with their CAH patients. Furthermore, most of the respondents who were already aware of the term learned about it through the internet; this issue warrants further exploration because unguided internet searches could potentially increase rates of misinformation and amplify feelings of stigmatization.

Over 75% of respondents felt that the term DSD has a negative effect on the CAH community. Review of optional comments from the survey showed that many respondents find the term DSD to be misleading and potentially stigmatizing. They expressed concern that friends and others who see the diagnosis of CAH associated with the term DSD might misunderstand and think they have a sexual disorder. Words used by survey participants in response to the term DSD include *horrific, offensive, humiliating, confusing, embarrassing, traumatizing, derogatory, demeaning, alarming,* and *sensationalistic*. The words “stigma” and “negative” were used over 100 times in comments by participants. Others remarked that calling CAH a DSD detracts from the more important medical issues related to adrenal insufficiency, especially since the term DSD refers only to females with classical CAH and not to patients with non-classical CAH or males.

Although the term DSD refers only to females with the classical form of CAH, males and non-classical patients are often mistakenly linked to the term. Because many patients have commented that the term DSD segregates the CAH community, the authors felt it was important to maintain cohesiveness and survey the CAH community as a whole. The results show that the non-DSD patients feel just as strongly about the term as classical females. There was no difference in opinion scores between classical females and non-DSD patients with CAH. The average scores for the two groups were 2.0 and 2.1, scores that correspond with “dislike” (Table 1). Likewise, there was no difference between U.S. and non-U.S. respondents, between surgical and non-surgical patients, or between adult patients who took the survey themselves and parents who took the survey. These results indicate a consistent attitude across different subgroups within the CAH community.

Within the same context, most CAH patients prefer not to be associated with the term DSD. In fact, more than half of those surveyed said they would not choose to receive care from centers or participate in research studies that use the term DSD. As a consequence, the current nomenclature may pose a barrier for certain patients to seek expert care in a specialized DSD center or to participate in related research.

As the response rate from those surveyed was only 29%, the results from this survey are not necessarily representative of all patients with 21OHD nor can they be extrapolated to other conditions under DSD. Further investigation into the views of other patient populations under DSD would augment our understanding of how this nomenclature is perceived. It would also be worthwhile to survey medical professionals to determine whether they accept the new terminology and use it to communicate with their patients.

Another consideration when interpreting these results is the potential for self-selection bias; it is possible that only patients and parents who already had negative feelings about the nomenclature participated in the survey. However, the majority of respondents had never heard the term DSD prior to this survey; hence, the results seem to reflect unbiased initial reactions rather than preconceived notions.

## Conclusions

This study provides the first comprehensive survey revealing the perception of CAH patients towards an extremely sensitive topic. Evaluation of the views of nearly 600 CAH patients and parents indicates that the majority are dissatisfied with the term DSD. As the term has become more widely utilized in the literature, internet, research proposals and patient care centers, it is important to consider the impact of this nomenclature on affected individuals and families. Our results highlight the challenges within the field of DSD to reach a consensus regarding a sensitive topic and to bridge the gap between current medical practice and patient satisfaction. It is the authors’ belief that reconsideration of the current nomenclature and ongoing dialogue between the medical community and patients will eventually lead to removal of stigmatization, better management protocols and improved outcomes.

## Abbreviations

DSD: Disorder(s) of sex development
CAH: Congenital adrenal hyperplasia
21OHD: 21-hydroxylase deficiency
CARES: **C**ongenital **A**drenal hyperplasia **R**esearch **E**ducation and **S**upport
